# Knockdown of Stanniocalcin-1 inhibits growth and glycolysis in oral squamous cell carcinoma cells

**DOI:** 10.1515/biol-2022-0907

**Published:** 2024-07-24

**Authors:** Chanyuan Wang, Jianpei Hu, Lijian Wang

**Affiliations:** Graduate School, Zhejiang University of Chinese Medicine, Hangzhou, Zhejiang, 310053, China; Department of Stomatology, Lishui People’s Hospital, Liandu District, Lishui, Zhejiang, 323000, China; Department of Stomatology, Lishui People’s Hospital, No. 15 Dazhong Street, Liandu District, Lishui, Zhejiang, 323000, China

**Keywords:** oral squamous cell carcinoma, Stanniocalcin-1, growth, glycolysis, PI3K/Akt pathway

## Abstract

Oral squamous cell carcinoma (OSCC) is the most common malignancy among head and neck squamous cell carcinomas. Targeted therapy plays a crucial role in the treatment of OSCC. However, new and more targets are still needed to develop. Stanniocalcin-1 (STC-1) is a glycoprotein hormone that affects the progression of cancers. However, the potential role of STC-1 in OSCC progression remains to be explored. Here, we aimed to elucidate the role of STC-1 in OSCC. We revealed that STC-1 was highly expressed in OSCC tissues and is correlated with poor patient prognosis. Knockdown of STC-1 significantly suppressed the growth of OSCC cells and restrained glycolysis by reducing glucose consumption, ATP production, and lactate levels. Mechanistically, STC-1 ablation inhibited the PI3K/Akt pathway, reducing the phosphorylation levels of PI3K and Akt. In conclusion, STC-1 depletion restrained OSCC cell growth and glycolysis via PI3K/Akt pathway and has the potential to serve as a therapeutic target for OSCC.

## 1 Introduction

Oral squamous cell carcinoma (OSCC) is the most common type of malignancy in head and neck squamous cell cancer [1]. Despite extensive research efforts to identify pathogenic factors and develop new treatments, the overall prognosis remains poor [2]. The occurrence of metastasis is a major factor contributing to poor survival in patients with OSCC and has become a prominent research focus in studies investigating possible potential treatments for OSCC [3]. Targeted therapy plays a crucial role in the treatment of OSCC by targeting specific molecular markers or signaling pathways to inhibit tumor growth and spread. For example, targeted drugs against the EGFR, such as Erlotinib and Cetuximab, have been used in the treatment of OSCC and have shown some efficacy [4,5]. However, new and more targets are still needed to develop.

Tumor cells primarily rely on oxidative phosphorylation for energy, but their growth is predominantly fueled by glycolysis, a process known as aerobic glycolysis, which is a fundamental biochemical characteristic of cancer cells [6,7]. Targeting cancer cell glycolysis is considered a promising approach for therapeutic interventions [6,7]. The PI3K/Akt pathway is vital in various cellular functions such as movement, autophagy, and cancer progression, and it is also critical in regulating metabolic adaptations that support cell growth [8]. Akt is considered a significant driver of the glycolytic phenotype in tumor cells, making them dependent on glycolysis for survival [9]. Akt enhances glucose uptake and glycolysis by increasing the expression of GLUT1, activating glycolytic enzymes, and regulating the expression, activity, as well as the interaction of HK with mitochondria [9].

Stanniocalcin-1 (STC-1) is a glycoprotein hormone implicated in the progression of various cancers [10]. STC-1 expression has been found during many developmental and pathophysiological processes in mammals [11,12]. Recent studies have shown that STC-1 promotes tumor angiogenesis, glycolysis, and metastasis in several malignancies, including breast, lung, and gastric cancers. Knockdown of STC-1 inhibits glycolysis in prostate cancer [13,14]. STC-1 promotes tumor angiogenesis by upregulating VEGF in gastric cancer cells [15]. Knockdown of STC-1 inhibits glycolysis in prostate cancer [16]. STC-1 enhances the stem-like characteristics of glioblastoma cells by binding to and activating NOTCH1 [17]. However, the potential role of STC-1 in OSCC progression remains to be explored.

In this study, we aimed to elucidate the role of STC-1 in OSCC and explore the therapeutic potential of targeting STC-1 in OSCC treatment. We found that STC1 is highly expressed in oral squamous cell cancer cells, and STC1 knockdown can inhibit OSCC cell growth and glycolysis, which is related to the regulation of the PI3K/Akt pathway.

## 2 Materials and methods

### 2.1 Bioinformatics

Transcriptome data for STC-1 in OSCC were obtained from The Cancer Genome Atlas (TCGA) database using the Genomic Data Commons Data Portal. Differential expression analysis was conducted between normal oral tissues (*n* = 40) and OSCC tissues (*n* = 520) using DESeq2 software. Statistical significance was evaluated using an adjusted *p*-value threshold of <0.05. Survival analysis was performed using data from the UALCAN database (https://ualcan.path.uab.edu/), where patients were categorized into high and low STC-1 expression groups based on median STC-1 expression levels. Kaplan–Meier survival curves were generated, and the log-rank test was used to determine significance (*p* < 0.05).

### 2.2 Cell culture and transfection

Human OSCC cell lines, including HSC-3 and CAL-27, and normal oral keratinocyte cell line HOK were purchased from ATCC. HOK, HSC-3, and CAL-27 cells were cultured with the DMEM (Gibco, USA) containing 10% FBS (Gbico, USA).

For STC-1 knockdown, two types of STC-1 shRNAs (sh-STC-1#1 and sh-STC-1#2) were constructed by our lab and cloned into a plasmid vector obtained from Addgene (USA). HSC-3 and CAL-27 cells were transfected with shRNA constructs using Lipofectamine 2000 (Invitrogen, USA) according to the manufacturer’s instructions. Control cells were transfected with a scrambled shRNA (sh-NC).

### 2.3 Immunoblotting

Samples were separated by sodiumdodecyl sulphate-polyacrylamide gel electrophoresis and transferred to polyvinylidene fluoride membranes. The membranes were blocked with 5% milk for 2 h, and then, the primary antibodies were added. Primary antibodies including anti-STC-1 (Abcam, ab2910; 1:1,000), HK2 (Abcam, ab209847; 1:1,000), LDHA (Abcam, ab52488; 1:500), Akt (Abcam, ab8805; 1:1,000), p-Akt (Abcam, ab38449; 1:500), PI3K (Abcam, ab302958; 1:1,000), p-PI3K (Abcam, ab278545; 1:500), and β-actin (Abcam, ab8226; 1:3,000, Cambridge, UK) antibodies and incubated with membranes for overnight at 4°C. Then, the membranes were incubated with secondary antibodies 1 h at room temperature. Protein expression was quantified using ImageJ software.

### 2.4 Cell counting kit-8 (CCK-8) assay

HSC-3 and CAL-27 cells were plated and maintained for 24, 48, and 72 h. Cells were subsequently incubated with CCK-8 (C0038, Beyotime, China). Then, the OD450 value was detected.

### 2.5 Edu assay

HSC-3 and CAL-27 cells were incubated with Edu agent (Abcam) for 2 h and removed the agent, then photographed by a microscope (Zeiss, German). Edu-positive cells were photographed using a fluorescence microscope (Zeiss, Germany) and quantified as a percentage of total cells.

### 2.6 Glucose intake, lactate, and ATP production test

The glycolysis levels of cells were detected using the kits including glucose intake (ab136955), lactate production (ab65330), and cellular ATP levels (ab83355; Abcam, Cambridge, UK).

### 2.7 Statistics

GraphPad 8.0 software was used and performed. Data were represented as mean ± standard deviation. *p* < 0.05 was thought as significant. Statistical significance was evaluated using Student’s *t*-test or analysis of variance, and *p* < 0.05 was considered statistically significant.

## 3 Results

### 3.1 STC-1 was highly expressed in human OSCC tissues and cells

**Figure 1:**
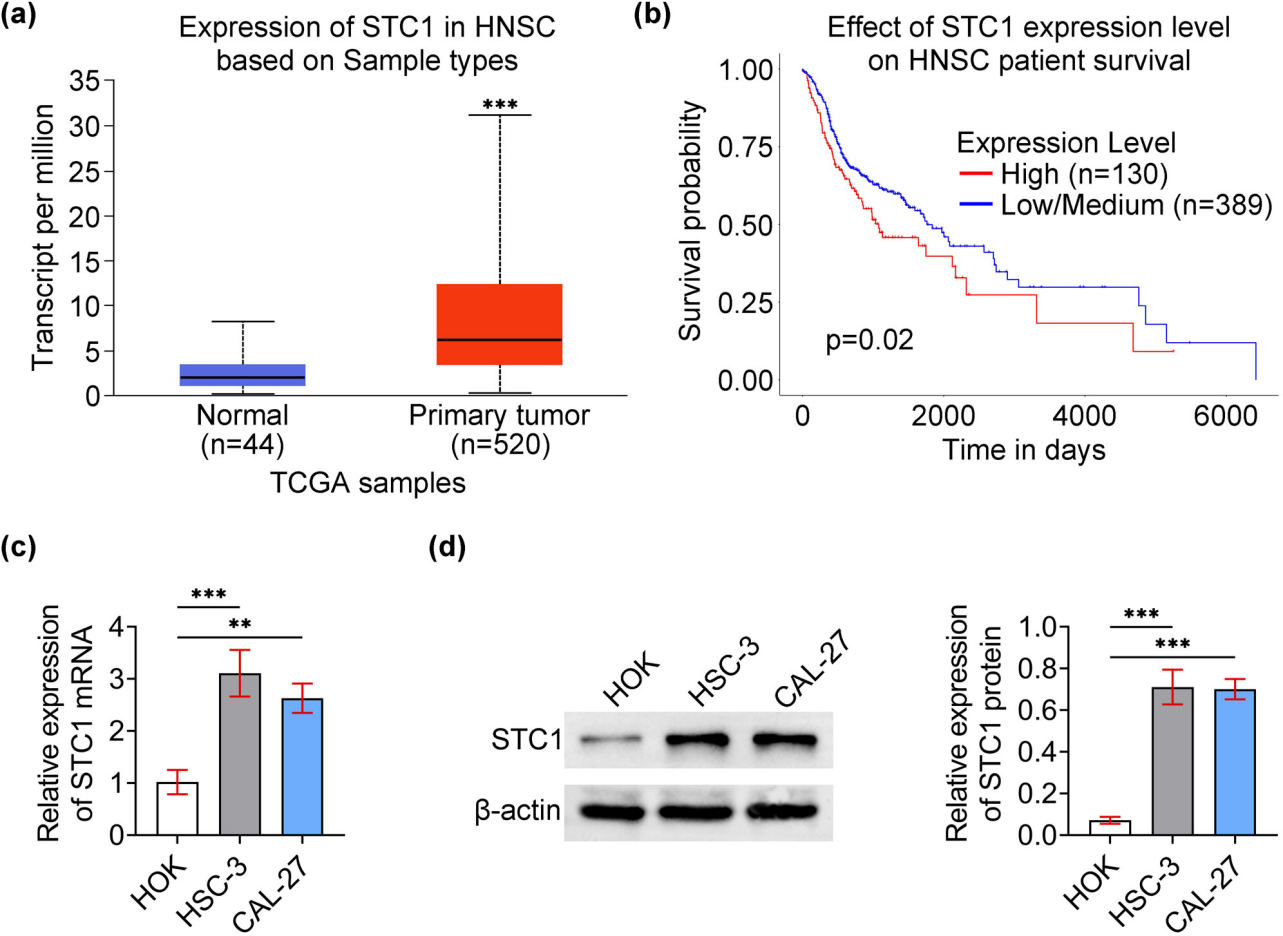
STC-1 was highly expressed in human OSCC tissues and cells. (a) TCGA database showed the TPM of STC-1 in 40 normal tissues and 520 OSCC tissues. (b) Ualcan database showed the correlation between STC-1 expression and the survival of OSCC patients (*p* = 0.02). (c) qPCR assays showed the mRNA levels of STC-1 in HOK, HSC-3, and CAL-27 cell lines. (d) Immunoblot assays showed the expression of STC-1 in HOK, HSC-3, and CAL-27 cell lines. **p < 0.01, ***p < 0.001, HSC3/CAL-27 vs HOK.

To detect the role of STC-1 in OSCC, we first detected its expression in OSCC tissues. Interestingly, we noticed the low transcripts per million (TPM) value of STC-1 in OSCC tissues (Figure 1a). Further through the Ualcan database, we noticed that the expression of STC-1 was correlated with the poor prognosis (survival) of patients with OSCC (*p* = 0.02, Figure 1b). Subsequently, we detected the expression of STC-1 in OSCC cell lines, including HSC-3 and CAL-27 cells, and normal oral keratinocyte cell line HOK cells. Through quantitative polymerase chain reaction (qPCR) assays, we revealed the high mRNA levels of STC-1 in OSCC cell lines (Figure 1c). Similarly, immunoblot assays confirmed that STC-1 was upregulated in HSC-3 and CAL-27 cells compared to HOK cells (Figure 1d). Therefore, STC-1 was highly expressed in OSCC tissues and cells.

### 3.2 The depletion of STC-1 suppressed the growth of OSCC cells

**Figure 2:**
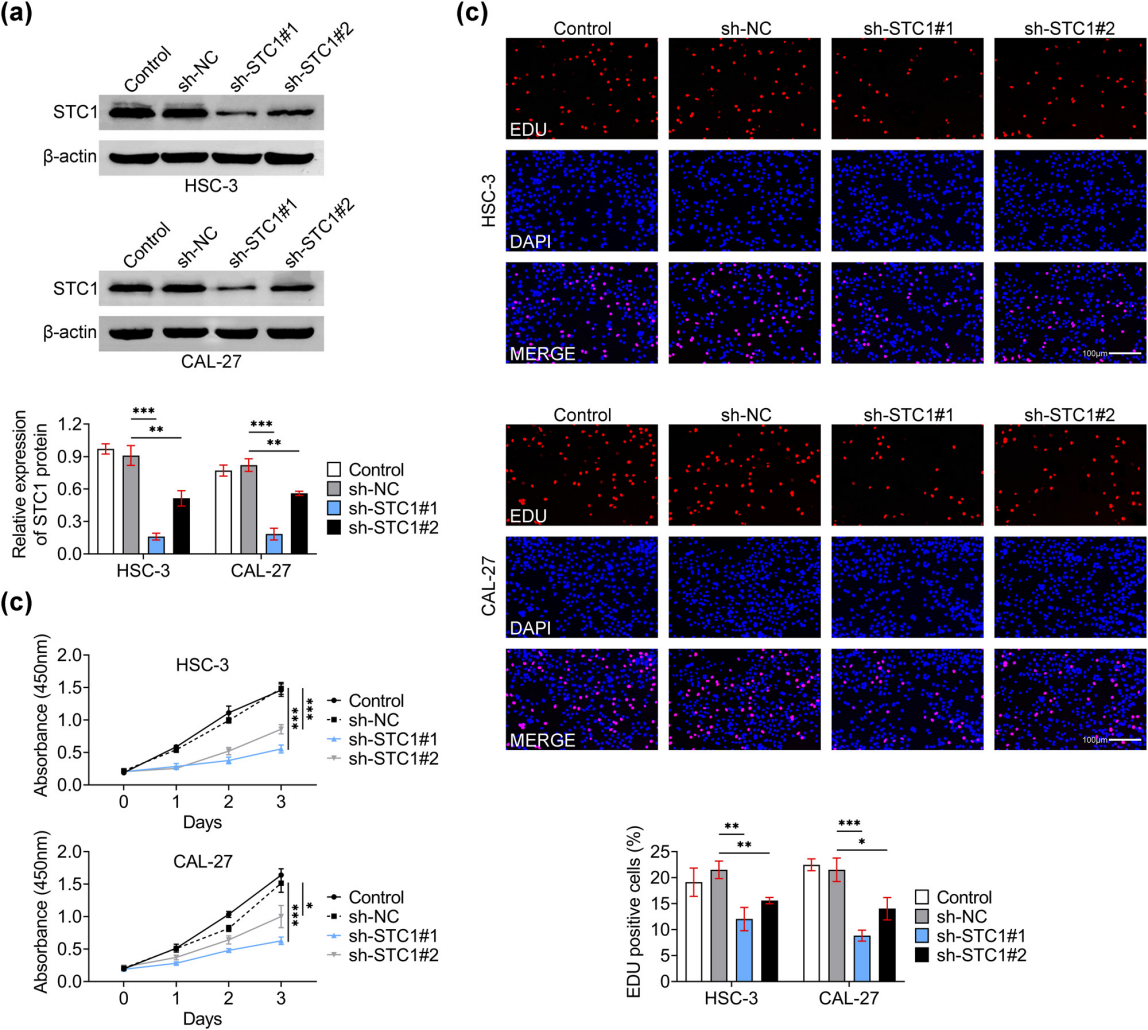
The depletion of STC-1 suppressed the growth of OSCC cells. (a) Immunoblot assays showed the expression of STC-1 in HSC3 (up) and CAL27 (down) cells upon the transfection of sh-NC, sh-STC-1#1, and sh-STC-1#2 for 24 h. The relative STC-1 expression was quantified. (b) CCK-8 assays showed the growth of HSC-3, and CAL-27 cells upon the transfection of sh-NC, sh-STC-1#1, and sh-STC-1#2 for 24, 48, and 72 h. OD450 value was measured. (c) Edu assays showed the growth degree of HSC-3, and CAL-27 cells upon the transfection of sh-NC, sh-STC-1#1, and sh-STC-1#2 for 24 h. The percentage of Edu-positive cells was quantified. Scale bar, 100 μm. Red panel indicates Edu. *p < 0.05, **p < 0.01, ***p < 0.001, sh-STC-1 vs sh-NC. NC, negative control.

We next used shRNAs to confirm the role of STC-1 in OSCC cells including HSC-3 and CAL-27 cells and the growth of OSCC cells. STC-1 was downregulated in these cells by the transfection of STC-1 shRNAs, including sh-STC-1#1 and sh-STC-1#2. Through Immunoblot assays, we noticed that the expression of STC-1 was significantly decreased upon the transfection of these shRNAs (Figure 2a). Interestingly, CCK-8 assays confirmed that the downregulation of STC-1 restrained the growth of HSC-3 and CAL-27 cells, with the decreased OD450 value at 1, 2, and 3 days (Figure 2b). Consistently, Edu assays showed that the percentage of Edu-positive cells was decreased upon the knockdown of STC-1 in HSC-3 and CAL-27 cells, suggesting the suppression of cell growth (Figure 2c). Collectively, STC-1 knockdown suppressed the growth of OSCC cells.

### 3.3 STC-1 ablation restrained the glycolysis of OSCC cells

**Figure 3:**
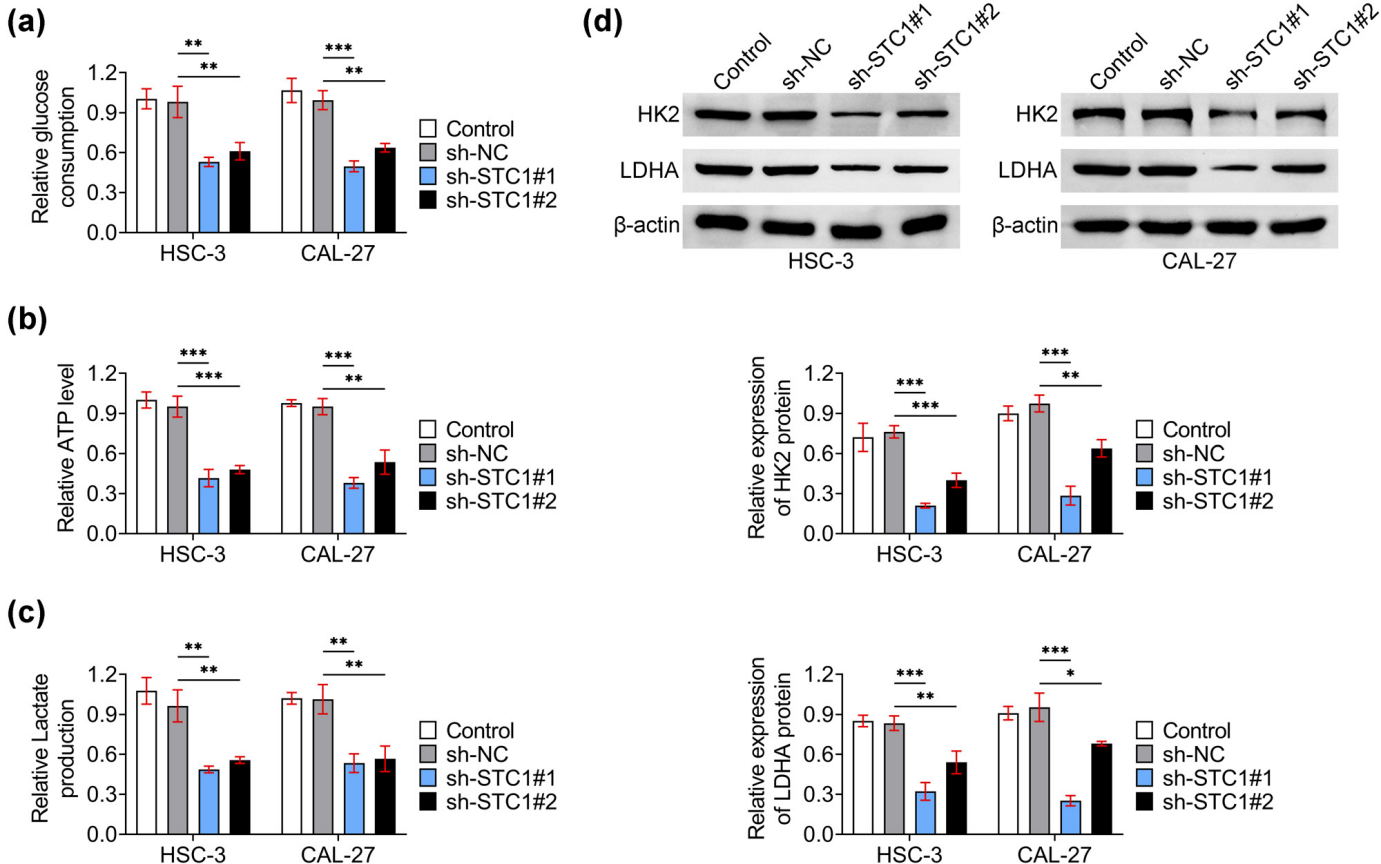
STC-1 ablation restrained the glycolysis of OSCC cells. (a) The relative glucose consumption of HSC-3, and CAL-27 cells upon the transfection of sh-NC, sh-STC-1#1, and sh-STC-1#2 for 24 h was measured. (b) The ATP levels of HSC-3, and CAL-27 cells upon the transfection of sh-NC, sh-STC-1#1, and sh-STC-1#2 for 24 h was measured by the ATP detection kit. (c) The relative Lactate production of HSC-3, and CAL-27 cells upon the transfection of sh-NC, sh-STC-1#1, and sh-STC-1#2 for 24 h was measured by the kit. (d) Immunoblot showed the expression of HK2 and LDHA in HSC-3 and CAL-27 cells upon the transfection of sh-NC, sh-STC-1#1, and sh-STC-1#2 for 24 h. *p < 0.05, **p < 0.01, ***p < 0.001, sh-STC-1 vs sh-NC. NC, negative control.

Then, we detected the effects of STC-1 in HSC-3 and CAL-27 cells on the cell glycolysis. By the use of the glucose consumption kits, we noticed that knockdown of STC-1 suppressed the glucose consumption in HSC-3 and CAL-27 cells (Figure 3a). Furthermore, we noticed that STC-1 knockdown suppressed the production of ATP levels in these OSCC cells (Figure 3b). Our results further confirmed that the lactate production was suppressed upon STC-1 knockdown in HSC-3 and CAL-27 cells (Figure 3c). Consistently, we performed the Immunoblot assays, and the data confirmed that STC-1 knockdown restrained the expression of HK2 and LDHA, two markers of glycolysis, in OSCC cells (Figure 3d). Therefore, STC-1 knockdown restrained the glycolysis of OSCC cells.

### 3.4 STC-1 knockdown suppressed the PI3K/Akt pathway in OSCC cells

**Figure 4:**
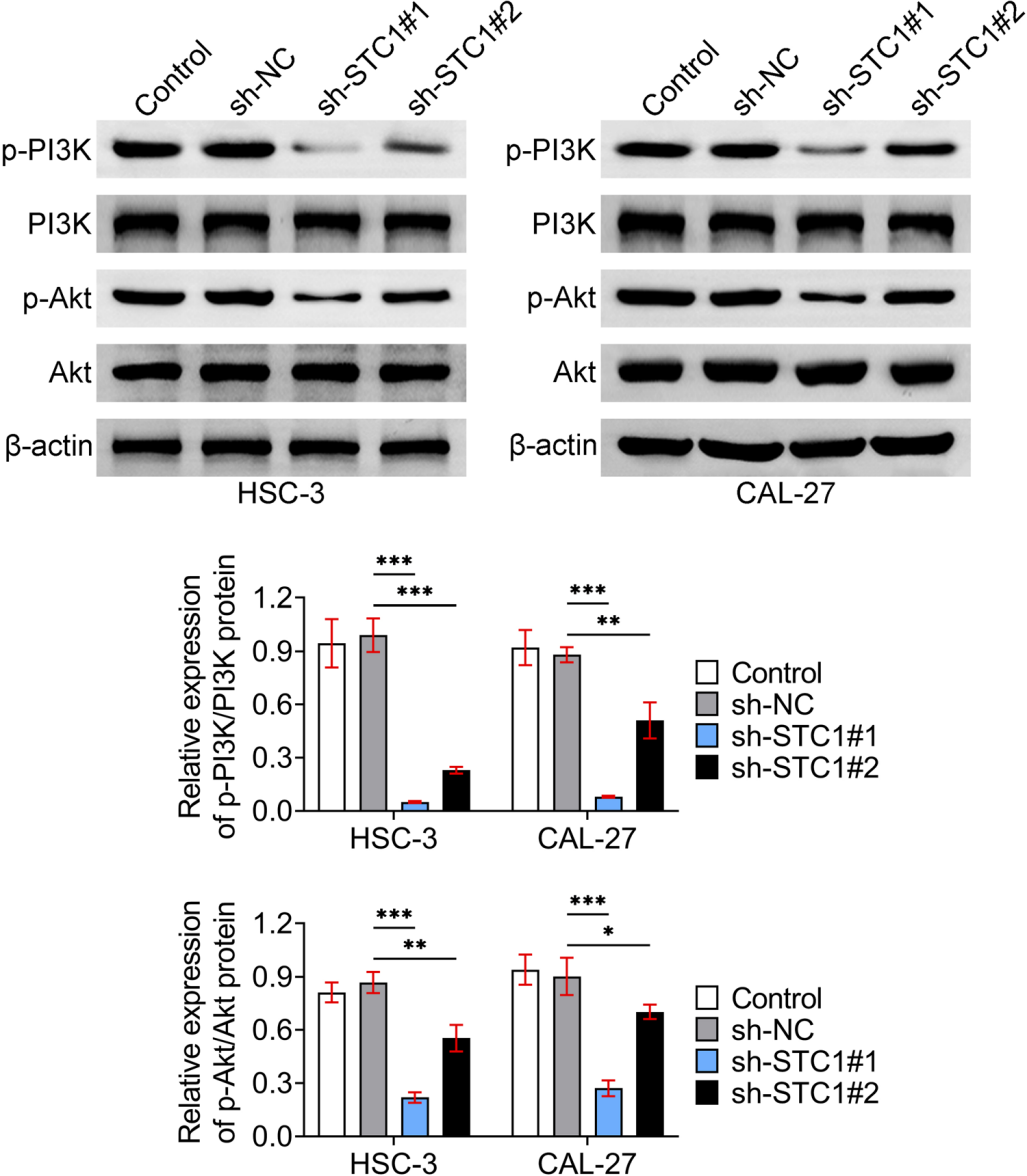
STC-1 knockdown suppressed the PI3K/Akt pathway in OSCC cells. Immunoblot showed the expression and phosphorylation levels of PI3K and Akt in HSC-3, and CAL-27 cells upon the transfection of sh-NC, sh-STC-1#1, and sh-STC-1#2 for 24 h was measured by the kit. The relative phosphorylation levels were quantified. *p < 0.05, **p < 0.01, ***p < 0.001, sh-STC-1 vs sh-NC. NC, negative control.

Finally, the possible mechanism underlying STC-1 knockdown suppressing OSCC progression was investigated. Through Immunoblot, we detected the phosphorylation levels of PI3K and Akt in both HSC-3 and CAL-27 cells. Interestingly, we noticed that STC-1 ablation suppressed the phosphorylation of both PI3K and Akt in HSC-3 and CAL-27 cells, suggesting the suppression of the PI3K/Akt pathway (Figure 4). Therefore, we believe that STC-1 knockdown suppressed the PI3K/Akt pathway in OSCC cells.

## 4 Discussion

OSCC remains a significant global health challenge due to its high incidence and mortality rates [4]. Current treatments for OSCC include surgery, radiotherapy, and chemotherapy, but the outcomes are often not satisfactory, with a high rate of recurrence and metastasis [1]. There is an urgent need for the development of targeted therapies for OSCC, which require the identification and validation of specific molecular targets [2]. The selection of these targets is crucial for improving therapeutic efficacy, reducing side effects, and improving the overall survival and quality of life of OSCC patients [5]. Key areas of focus include EGFR inhibitors like cetuximab and erlotinib, immunotherapies targeting PD-1/PD-L1 pathways such as pembrolizumab and nivolumab, and exploration of other molecular targets like VEGF, HER2, and the PI3K/AKT/mTOR pathway [18]. Efforts are also underway to personalize treatment based on individual tumor profiles [18]. However, the integration of targeted therapies into standard OSCC treatment protocols is still in the developmental stages and requires further research. Interestingly, we here indicated that STC-1 is highly expressed in OSCC cells, and knocking down STC-1 can inhibit OSCC cell growth and glycolysis. Therefore, we believed that STC-1 has the potential to serve as a promising target of OSCC.

STC-1 is a glycoprotein hormone involved in calcium and phosphate homeostasis, cellular protection against stress, angiogenesis, and inflammation [10,19]. The relationship between STC-1 and cancer has become a focus of research, with elevated expression of STC-1 observed in various cancers, including breast cancer, lung cancer, and ovarian cancer [14,20,21]. Studies have shown that STC-1 may promote tumor growth by activating signaling pathways such as PI3K/Akt and MAPK, facilitate tumor metastasis by affecting the degradation of the extracellular matrix, regulate the tumor microenvironment by promoting angiogenesis, and inhibit tumor cell apoptosis leading to chemotherapy resistance [22,23]. These findings underscore the multifunctional role of STC-1 in tumor biology and highlight the therapeutic potential of targeting STC-1 across various cancers. Strategies targeting STC-1 may help inhibit tumor growth and metastasis and improve chemotherapy efficacy. However, the specific mechanisms of STC-1 in cancer still require further investigation. Herein, our results confirmed that STC-1 ablation inhibited OSCC cell growth and glycolysis. However, the precise mechanism needs further study.

OSCC is closely associated with altered glucose metabolism, particularly an increase in glycolysis, a phenomenon known as the Warburg effect. This metabolic reprogramming is a hallmark of cancer cells, including those in OSCC, and is characterized by a preference for glycolysis over oxidative phosphorylation for energy production, even in the presence of oxygen [24,25]. Interestingly, we confirmed that STC-1 could serve as a key regulator in mediating glycolysis in OSCC cells. We believed that STC-1 may affect OSCC progression via mediating glycolysis.

The PI3K/Akt pathway is a critical regulator of cancer metabolism, and our data confirmed that STC-1 ablation suppresses this pathway in OSCC cells. Previous studies have demonstrated that inhibiting the PI3K/Akt pathway can significantly reduce glycolysis and tumor progression in colorectal cancer and nasopharyngeal carcinoma [26,27]. Activation of this pathway enhances glucose uptake by increasing the translocation of glucose transporters like GLUT1, upregulates key glycolytic enzymes such as hexokinase, phosphofructokinase, and pyruvate kinase, and inhibits apoptosis, allowing for sustained cancer cell proliferation [26]. In addition, Akt regulates HIF-1α, which further enhances glycolysis under hypoxic conditions, and alters the localization of metabolic enzymes like hexokinase to the mitochondria, increasing glycolytic flux [28]. Therefore, targeting the PI3K/Akt pathway is a potential therapeutic strategy to disrupt the altered energy metabolism in cancer cells. Notably, this pathway plays a crucial role in the development of OSCC by influencing cell proliferation, survival, angiogenesis, and metastasis [29]. Its activation is associated with resistance to conventional therapies, making it a potential target for therapeutic intervention. Our findings confirmed that STC-1 ablation inhibits the PI3K/Akt pathway, thereby inhibiting OSCC cell growth and glycolysis.

Overall, our findings, combined with those from other studies, highlight the potential of STC-1 as a promising therapeutic target in OSCC and other cancers. Future studies will focus on developing specific inhibitors targeting STC-1 and evaluating their efficacy in clinical settings.
