# Corrigendum to “Knockdown of Stanniocalcin-1 inhibits growth and glycolysis in oral squamous cell carcinoma cells”

**DOI:** 10.1515/biol-2025-1334

**Published:** 2026-05-14

**Authors:** Chanyuan Wang, Jianpei Hu, Lijian Wang

**Affiliations:** Graduate School, Zhejiang Chinese Medical University, Hangzhou, Zhejiang Province, 310053, China; Department of Stomatology, Lishui People’s Hospital, Lishui, Zhejiang Province, 323000, China

In the published manuscript, “Knockdown of Stanniocalcin-1 inhibits growth and glycolysis in oral squamous cell carcinoma cells. Open Life Sciences. 2024, 19(1): 20220907. https://doi.org/10.1515/biol-2022-0907,” the first institutional affiliation of the authors Chanyuan Wang was incorrectly given as “ZhejiangUniversity of Chinese Medicine” The correct affiliation is “Zhejiang Chinese Medical University”.

